# The BH3-only proteins BIM and PUMA are not critical for the reticulocyte apoptosis caused by loss of the pro-survival protein BCL-XL

**DOI:** 10.1038/cddis.2017.304

**Published:** 2017-07-06

**Authors:** Alex RD Delbridge, Brandon J Aubrey, Craig Hyland, Jonathan P Bernardini, Ladina Di Rago, Jean-Marc Garnier, Guillaume Lessene, Andreas Strasser, Warren S Alexander, Stephanie Grabow

**Affiliations:** 1 The Walter and Eliza Hall Institute of Medical Research, Melbourne, VIC, Australia; 2 Department of Medical Biology, University of Melbourne, Melbourne, VIC, Australia; 3 Department of Pharmacology and Therapeutics, University of Melbourne, Melbourne, VIC, Australia

## Abstract

Anaemia is a major global health problem arising from diverse causes and for which improved therapeutic strategies are needed. Erythroid cells can undergo apoptotic cell death and loss of pro-survival BCL-XL is known to trigger apoptosis during late-stage erythroid development. However, the mechanism by which loss or pharmacological blockade of BCL-XL leads to erythroid cell apoptosis remains unclear. Here we sought to identify the precise stage of erythropoiesis that depends on BCL-XL. We also tested whether deficiency of BIM or PUMA, the two main pro-apoptotic antagonists of BCL-XL, could prevent reticulocyte death and anaemia caused by BCL-XL loss. Using an *in vivo* mouse model of tamoxifen-inducible *Bclx* gene deletion and *in vitro* assays with a BCL-XL-selective inhibitor, we interrogated each stage of erythrocyte differentiation for BCL-XL dependency. This revealed that reticulocytes, but not orthochromatic erythroblasts, require BCL-XL for their survival. Surprisingly, concurrent loss of BIM or PUMA had no significant impact on the development of anemia following acute BCL-XL deletion *in vivo*. However, analysis of mixed bone marrow chimaeric mice revealed that loss of PUMA, but not loss of BIM, partially alleviated impaired erythropoiesis caused by BCL-XL deficiency. Insight into how the network of pro-survival and pro-apoptotic proteins works will assist the development of strategies to mitigate the effects of abnormal cell death during erythropoiesis and prevent anaemia in patients treated with BCL-XL-specific BH3-mimetic drugs.

Anaemia affects over 1.5 billion people world-wide^1^ and is a major cause of morbidity that requires improved therapeutic interventions. Underlying causes for anaemia are diverse and include haemoglobinopathies, red cell enzyme disorders, nutritional deficiencies, cytoskeletal abnormalities and autoimmune diseases. Furthermore, anaemia is a frequent toxicity associated with cancer therapy, including both conventional chemotherapy as well as emerging targeted therapies, such as some of the BH3-mimetics.^2, 3^ Understanding the requirements for normal erythroid development and survival will help develop improved treatment strategies to support erythropoietic function in patients.

In the adult, mature red blood cells derive from haematopoietic stem cells. Erythropoiesis proceeds through several differentiation steps whereby the potential for alternate blood cell lineages is progressively lost and immature progenitors expand in number to facilitate production of sufficient mature red blood cells, with half-lives spanning 100–120 days in humans and 8–20 days in mice.^4, 5, 6, 7^ Should bone marrow erythropoiesis become compromised – due to bone marrow failure/dysplasia or infiltration, or excessive loss/destruction of peripheral red blood cells – extramedullary erythropoiesis can occur in the spleen to facilitate enhanced erythrocyte production in mice. Robust pro-survival signalling is essential to prevent excessive apoptotic cell death during erythropoiesis and the maintenance of adequate red blood cell numbers. Expression analysis has identified the pro-survival gene *Bclx* as particularly highly expressed in the erythroid lineage,^8^ suggestive of its critical importance for countering apoptosis during erythropoiesis. Aberrant apoptosis of red blood cells and erythroid progenitors is a common feature of chronic anaemias and is likely to contribute to their severity. For example, thalassaemia patients have elevated numbers of early precursor cells in the bone marrow but abnormally high rates of apoptosis in more mature progenitors,^9^ whereas in anaemia of chronic disease, elevated interferon-*γ* levels are associated with increased apoptosis of erythroid progenitors and inversely correlated with reticulocyte numbers and haemoglobin levels.^10^ Aberrant apoptosis is also a key feature of Diamond-Blackfan Anaemia where ribosomal stress drives abnormal TP53 activation and cell death.^11^ TP53 is known to regulate the expression of several key initiators of apoptosis, including *Puma, Noxa* and *Bax*.^12, 13, 14, 15^

The intrinsic (also called ‘BCL-2-regulated’, ‘stress’ or ‘mitochondrial’) apoptotic pathway is regulated by three major classes of proteins.^16, 17^ The pro-survival proteins (e.g., BCL-2, BCL-XL, MCL-1) antagonise the pro-apoptotic effectors BAX and BAK. Upon stress stimuli pro-apoptotic BH3-only proteins, such as BIM and PUMA, are induced transcriptionally or post-transcriptionally in a cell type and stimulus-specific manner, and initiate apoptosis by displacing BAX/BAK from their restraint by the pro-survival proteins and possibly also by direct activation of BAX/BAK.^18^ Activated BAX and BAK mediate disruption of the outer mitochondrial membrane. This represents the ‘point of no return’ for the cell, triggering activation of the caspase cascade, resulting in proteolysis of many substrates and cellular demolition.^19^

In accordance with their roles in apoptosis signalling, loss of pro-apoptotic BIM or combined loss of BAX and BAK result in abnormal accumulation of diverse haematopoietic cell types,^20, 21^ whereas conversely loss of pro-survival MCL-1 results in bone marrow failure.^22, 23^ In the erythroid lineage BCL-XL is of critical importance. Germline deletion of *Bclx* results in embryonic death of mice at ~E13.5 in part owing to increased apoptosis of erythroid progenitors; interestingly, this death of erythroid progenitors could be rescued by concomitant loss of BIM.^24, 25^ Tissue-restricted deletion of *Bclx* results in severe anaemia, splenomegaly due to erythroblast accumulation, and thrombocytopenia.^26^ Loss or inhibition of BCL-XL (e.g., using the BH3-mimetics ABT-737 or ABT-263 ref. 27, 28), but not of BCL-2 (e.g., using ABT-199 ref. 29), results in thrombocytopenia in patients and mouse models and also anaemia in mice.^2, 24, 26, 27, 30, 31, 32, 33, 34^ It has yet to be established whether pharmacological inhibition of BCL-XL will also cause anaemia in patients. Published data demonstrate that both BAX and BAK must be removed to prevent anaemia caused by loss or drug mediated inhibition of BCL-XL, as they have largely overlapping roles in the execution of apoptosis.^26, 35^ It is, however, still unknown which BH3-only protein is responsible for the initiation of apoptosis in this context.

We defined the requirement for BCL-XL at various stages of adult erythropoiesis by using a discriminating flow cytometry method^36^ and a tamoxifen-inducible, acute *Bclx* gene deletion mouse model. Given that BIM is essential for the aberrant apoptosis of erythroid progenitors in embryonic mice caused by the absence of BCL-XL,^25^ we investigated the role of this pro-apoptotic BH3-only protein in adult erythropoiesis. We found that BCL-XL is critical for the survival of reticulocytes and that BIM is not essential for the anaemia that is caused by acute loss of BCL-XL, whereas pro-apoptotic PUMA has a minor role. These discoveries inform the development of strategies to alleviate anaemia caused, for example, by inherited mutations, infections or treatment with anti-cancer agents, including the new BH3-mimetic drugs.

## Results

### Acute loss of BCL-XL causes profound anaemia in adult mice owing to failure of erythropoiesis

To confirm and extend published data characterising the role of BCL-XL in erythroid cell survival,^24, 26, 30^ we generated mice bearing floxed *Bclx (Bcl2l1)* alleles that could be deleted in an inducible manner by tamoxifen-dependent CreERT2-recombinase activity. *Bclx*^*fl*^ mice^26^ were crossed with *RosaCreERT2*^*Ki*^ mice^37^ to generate *Bclx*^*fl/fl*^*;RosaCreERT2*^*Ki/+*^ compound mutant mice. In these mice, tamoxifen administration activates the latent CreERT2 recombinase to facilitate recombination of the floxed *Bclx* alleles, leading to loss of BCL-XL expression.

**Figure 1:**
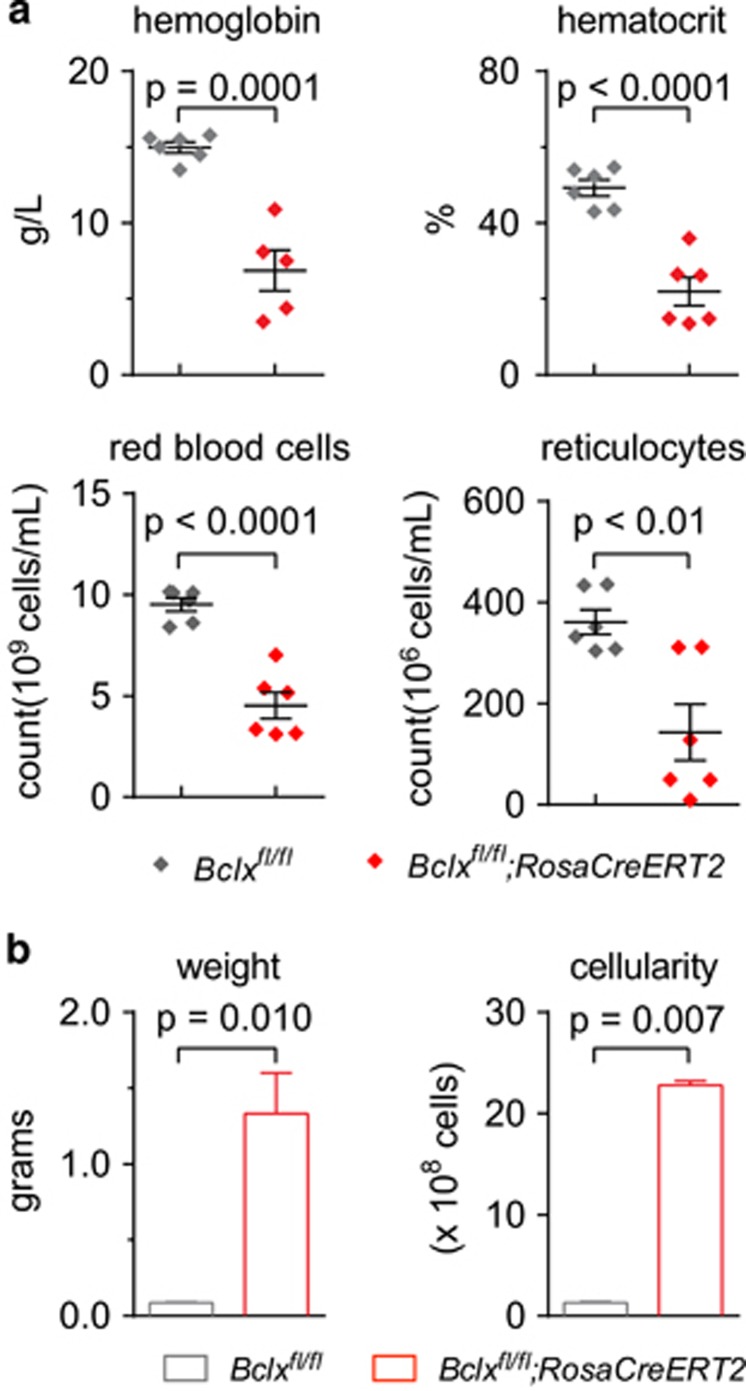
Acute loss of BCL-XL causes severe anaemia. *Bclx*^*fl/fl*^*;RosaCreERT2*^*Ki/+*^ mice and control *Bclx*^*fl/fl*^ mice were treated with tamoxifen (TAM) to induce *Bclx* gene deletion. After 1 month (**a**) blood analysis was performed and (**b**) spleen weight and cellularity were determined. *n*=5–6. Data are presented as mean±S.E.M. Significant *P-*values are shown, unpaired Students *t*-test

A cohort of *Bclx*^*fl/fl*^*;RosaCreERT2*^*Ki/+*^ mice and *Bclx*^*fl/fl*^ control mice were treated with tamoxifen at 8 weeks of age. At 1 month post treatment these adult mice were analysed to determine the effects of BCL-XL loss. Peripheral blood analysis confirmed previous reports,^26, 31^ with profound anaemia observed in the *Bclx*^*fl/fl*^*;RosaCreERT2*^*Ki/+*^ mice. Haemoglobin (*P*=0.0001) levels, haematocrit (*P*<0.0001) and reticulocyte as well as mature red blood cell numbers were all significantly reduced (Figure 1a). Conversely, both spleen weight and cellularity were increased in the *Bclx*^*fl/fl*^*;RosaCreERT2*^*Ki/+*^ mice (Figure 1b). In contrast, the tamoxifen-treated *Bclx*^*fl/fl*^ control mice retained normal blood and spleen cell counts.

A previous study reported that haemolysis was associated with BCL-XL deletion.^26^ We therefore examined the impact of inducible adult loss of BCL-XL on red blood cell turnover. Consistent with abnormal red blood cell destruction, we observed an increase in serum levels of unconjugated bilirubin in the tamoxifen-treated *Bclx*^*fl/fl*^*;RosaCreERT2*^*Ki/+*^ mice (Supplementary Figure 1A). Consistent with normal hepatic function, no increases in the levels of serum albumin, alkaline phosphatase, aspartate aminotransferase and gamma-glutamyl transferase (<4 U/l; data not shown) were observed. There was a marginal increase in alanine aminotransferase; however, the levels observed were below those indicative of significant liver damage (Supplementary Figure 1B). Hence, we conclude that liver dysfunction did not account for the accumulation of serum bilirubin. The serum haptoglobin levels were very low (<0.08 g/l; data not shown), consistent with abnormal red blood cell breakdown and release of haemoglobin. However, the lactate dehydrogenase levels were not increased, which suggests that red blood cell destruction may occur predominantly within the extra-vascular space (bone marrow and spleen) (Supplementary Figure 1B).

Blood films were prepared from tamoxifen-treated *Bclx*^*fl/fl*^*;RosaCreERT2*^*Ki/+*^and *RosaCreERT2*^*Ki/+*^ (control) mice to identify changes in red blood cells caused by loss of BCL-XL. Remarkably, almost complete disappearance of reticulocytes was seen by day 2 (Supplementary Figure 2A). This was preceded by morphologic changes in the reticulocytes of coarse basophilic stippling (Supplementary Figure 2B). There was no evidence of spherocytosis, red blood cell fragmentation (schistocytes) or specific changes typical of oxidative haemolysis (‘bite’ or ‘blister’ cells) following BCL-XL loss. Mild CRE recombinase toxicity was observed in control animals at day 5, with a minor reduction in reticulocytes. However, this effect was transient and haematopoietic reconstitution experiments revealed that transient CreERT2 activation exerted no long-term consequences (Supplementary Figure 2C).

Red blood cells constitute >95% of all blood cells and must be continuously replaced as they are lost or expended. Consequently, perturbation of bone marrow stem/progenitor cell activity may lead to the observed anaemia. Hence, we examined whether the reduction in red blood cells in the tamoxifen-treated *Bclx*^*fl/fl*^*;RosaCreERT2*^*Ki/+*^ mice was a consequence of defective bone marrow stem/progenitor cell activity. Flow cytometry was used to determine the proportions and numbers of stem and progenitor cell subsets in the bone marrow of these mice. This revealed that after tamoxifen treatment *Bclx*^*fl/fl*^*;RosaCreERT2*^*Ki/+*^ and *Bclx*^*fl/fl*^(control) mice had similar proportions and numbers of stem cells (LT-HSC, ST-HSC), multipotent progenitors (MPP) and committed progenitor cells (MEP, CMP, GMP) (Supplementary Figure 3A). As a functional readout of progenitor cells, we performed colony-formation assays with bone marrow and spleen cells in semi-solid agar. No significant differences were observed in the relative proportions of the various myeloid-committed colony-forming cells in the bone marrow and spleen between tamoxifen-treated *Bclx*^*fl/fl*^*;RosaCreERT2*^*Ki/+*^ and *Bclx*^*fl/fl*^(control) mice (Table 1). Therefore, we conclude that BCL-XL is dispensable for the survival of haematopoietic stem/progenitor cells in adult mice at steady state.

**Figure 2:**
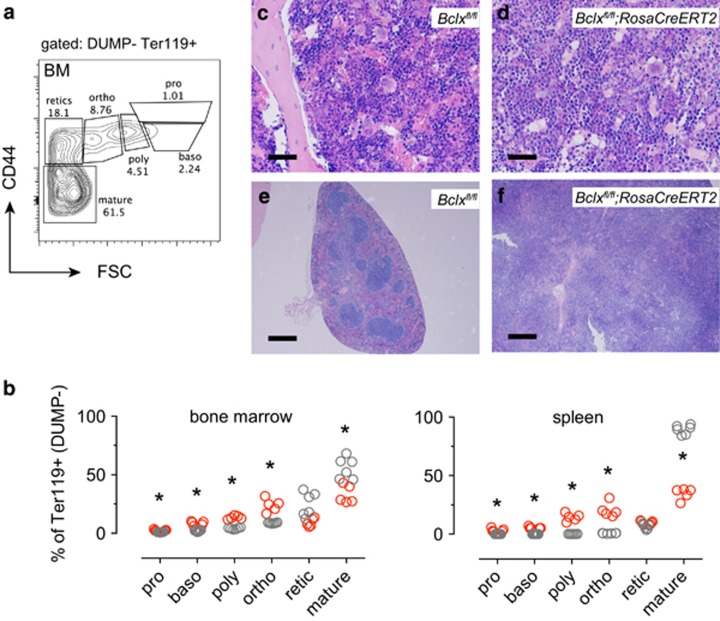
Acute deletion of BCL-XL results in an elevation of erythroid progenitors. *Bclx*^*fl/fl*^*;RosaCreERT2*^*Ki/+*^ mice and control *Bclx*^*fl/fl*^ mice were treated with tamoxifen (TAM) to induce *Bclx* gene deletion. Analysis of bone marrow and spleen was performed after 1 month. (**a**) Gating strategy using CD44 *versus* forward light scatter (FSC) to delineate defined erythropoietic stages; in order of increasing maturity: pro-erythroblasts (pro), basophilic erythroblasts (baso), polychromatic erythroblasts (poly), orthochromatic erythroblasts(ortho), reticulocytes (retic) and mature red blood cells (mature). (**b**) Erythroid subset analysis (%) in the bone marrow and spleen (*Bclx^fl/fl^;RosaCreERT2^Ki/+^* in red, and control *Bclx^fl/fl^* in grey). (**c–f**) Bone marrow cells harvested from *Bclx*^*fl/fl*^*;RosaCreERT2*^*Ki/+*^ mice (or control *Bclx*^*fl/fl*^ mice) 4 weeks after tamoxifen treatment were used to reconstitute lethally irradiated GFP^+^ recipient mice. *Bclx*^*fl/fl*^*;RosaCreERT2*^*Ki/+*^ bone marrow reconstituted mice developed anaemia after 3 weeks and were transfused with 250 *μ*l GFP^+^ blood. Histological analysis of sternum (**c, d**) and spleen (**e, f**) specimens collected from *Bclx*^*fl/fl*^*;RosaCreERT2*^*Ki/+*^ bone marrow reconstituted mice or control *Bclx*^*fl/fl*^ bone marrow reconstituted mice. Bone marrow sections (× 20 objective) scale bar=4 *μ*m, spleen sections (× 40 objective) scale bar=20 *μ*m. Data are representative of *n*=3 mice per genotype. (**b**) *n*=5–6, unpaired Students *t*-test, adjusted for multiple testing (Holm–Sidak); *P<0.002

We next wanted to define the precise stage at which red blood cell production is perturbed to identify the cause of the anaemia caused by loss of BCL-XL. One month after deletion of *Bclx*, red blood cell maturation was analysed by flow cytometry based on expression of TER119, CD44 and cell size (FSC) (ref. 36; Figure 2a). Following *Bclx* gene deletion we observed significantly decreased percentages of mature red blood cells, but not reticulocytes in the bone marrow with a corresponding increase in the percentages of the more immature precursors (Figure 2b). This phenomenon was mirrored in the spleen of the tamoxifen-treated *Bclx*^*fl/fl*^*;RosaCreERT2*^*Ki/+*^ mice, where normally only few immature erythroid progenitor cells are found. Colony-formation assays revealed a pronounced increase in the numbers of colony-forming units-erythroid (CFU-e) in the spleen of tamoxifen-treated *Bclx*^*fl/fl*^*;RosaCreERT2*^*Ki/+*^ mice compared with *Bclx*^*fl/fl*^mice, whereas no differences were observed in the bone marrow (Supplementary Figure 3B). These findings are consistent with erythropoietic insufficiency in the tamoxifen-treated *Bclx*^*fl/fl*^*;RosaCreERT2*^*Ki/+*^ mice, whereupon the spleen has been recruited as a site for extramedullary erythropoiesis in a reactive attempt to overcome the low numbers of mature red blood cells in the circulation.

Next, we harvested bone marrow cells from *Bclx*^*fl/fl*^*;RosaCreERT2*^*Ki/+*^ and *Bclx*^*fl/fl*^ mice one month following tamoxifen treatment and tested their ability to reconstitute the haematopoietic system of lethally irradiated recipient mice. The animals reconstituted with *Bclx*^*fl/fl*^*;RosaCreERT2*^*Ki/+*^ cells developed symptoms of erythropoietic failure within 3 weeks post-reconstitution. Blood transfusion was performed, however by 4 weeks post-reconstitution these mice reached ethical endpoint and were killed for analysis. Flow cytometric analysis gated on donor, non-transfused cells (GFP^−^) in the spleen and bone marrow revealed elevated proportions of immature erythroid progenitors with a decrease in the proportions of reticulocytes and mature red blood cells in the tamoxifen-treated *Bclx*^*fl/fl*^*;RosaCreERT2*^*Ki/+*^ mice (Supplementary Figure 4). Although no major architectural changes were observed in the bone marrow of these mice (Figures 2c and d), widespread disruption of splenic architecture was observed, consistent with compensatory erythropoiesis in this organ (Figures 2e and f). Mice reconstituted with *Bclx*^*fl/fl*^ bone marrow cells exhibited the expected erythroid subset proportions as well as normal splenic and bone marrow architecture.

### BCL-XL is essential for the survival of reticulocytes

**Figure 3:**
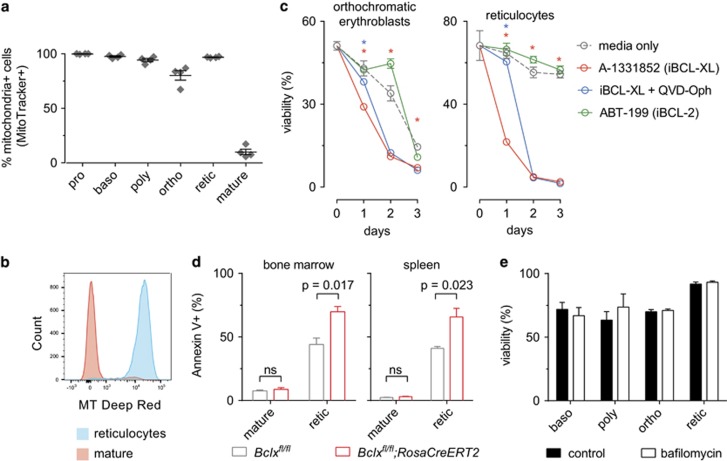
Loss of BCL-XL causes apoptotic death in reticulocytes. (**a**, **b**) Wildtype pro-erythroblasts (pro), basophilic erythroblasts (baso), polychromatic erythroblasts (poly), orthochromatic erythroblasts (ortho), reticulocytes (retic) and mature red blood cells (mature) were identified by flow cytometry by their FSC *versus* CD44 profile and the proportion of cells containing mitochondria was determined by with MitoTracker deep red staining. *n*=4; mean±S.E.M.; representative histogram shown. (**c**) Survival *in vitro* of FACS-sorted reticulocytes and orthochromatic erythroblasts harvested from wild-type mice. Cells were treated as indicated: A-1331852 (1 *μ*M, iBCL-XL), ABT-199 (1 *μ*M, iBCL-2), QVD-OPH (25 nM). Cell viability was quantified by forward *versus* side light scatter profile measured by FACS on days 1–3. Two-way ANOVA analysis with Tukey’s multiple comparisons test, comparison between time point means. Stars indicate significant differences: ‘red’ indicates medium *versus* iBCL-XL, ‘blue’ indicates iBCL-XL *versus* iBCL-XL plus QVD-OPH. *n*=4, mean±S.E.M. shown. (**d**) Annexin V staining was performed on reticulocytes and mature red blood cells isolated from the bone marrow and spleen of *Bclx*^*fl/fl*^*;RosaCreERT2*^*Ki/+*^ mice or control *Bclx*^*fl/fl*^ mice 24 h following tamoxifen administration. *n*=3, mean±S.E.M. shown. Significant differences indicated with *P-*values as shown, unpaired Students *t*-test, adjusted for multiple testing (Holm–Sidak). (**e**) Survival *in vitro* of FACS-sorted basophilic erythroblasts (baso), polychromatic erythroblasts (poly), orthochromatic erythroblasts (ortho), and reticulocytes (retic) from wild-type mice. Cells were treated with bafilomycin (0.1 *μ*M) and viability was determined by PI exclusion after 24 h and compared to untreated cells (control). No significant differences were observed. *n*=3, mean±S.E.M. shown

Analysis of mitochondrial content revealed that, as expected, 95% of reticulocytes contained mitochondria and only mature red blood cells were devoid of mitochondria (Figures 3a and b). Having confirmed that reticulocytes and erythroblasts contain the organelle required for the mitochondrial apoptotic pathway, we examined whether the loss of reticulocytes resulting from the absence of BCL-XL was caused by apoptosis. Wild-type reticulocytes were FACS-sorted and treated *in vitro* with BH3-mimetic drugs that specifically inhibit BCL-XL (A-1331852)^38^ or BCL-2 (ABT-199; used as a control) (Figure 3c). Treatment with A-1331852 resulted in the rapid death of reticulocytes, and this could be substantially reduced during the first 24 h by blocking caspase activation with QVD-OPH. In contrast, treatment with ABT-199 had no effect on reticulocyte viability (Figure 3c). Interestingly, the marked dependency on BCL-XL for survival was specific to reticulocytes, as orthochromatic erythroblasts were far less sensitive to A-1331852. Furthermore, following *Bclx* gene deletion a significant increase in the exposure of phosphatidyl-serine (detected by Annexin V staining) – an early apoptotic marker – was observed on reticulocytes, but not mature red blood cells, from the bone marrow and spleen (Figure 3d).

BCL-XL was implicated in mitochondria specific-autophagy (mitophagy) owing to the established role of Nip3-like protein-X (NIX) in the clearance of mitochondria from maturing red blood cells^39^ and the ability of BCL-XL to interact with the NIX homologue BNIP3.^40^ To investigate the involvement of mitophagy in the loss of reticulocytes caused by BCL-XL deletion, cell viability was measured following treatment of erythroid progenitors with the autophagy inhibitor – bafilomycin – that prevents fusion between autophagosomes and lysosomes.^41^ Bafilomycin had no impact on erythroid precursor survival (Figure 3e), consistent with the notion that autophagy plays no role in the loss of reticulocytes caused by BCL-XL deletion.

### Hematopoietic-specific loss of BCL-XL provokes anaemia in adult mice that cannot be alleviated by concomitant loss of BIM or PUMA

**Figure 4:**
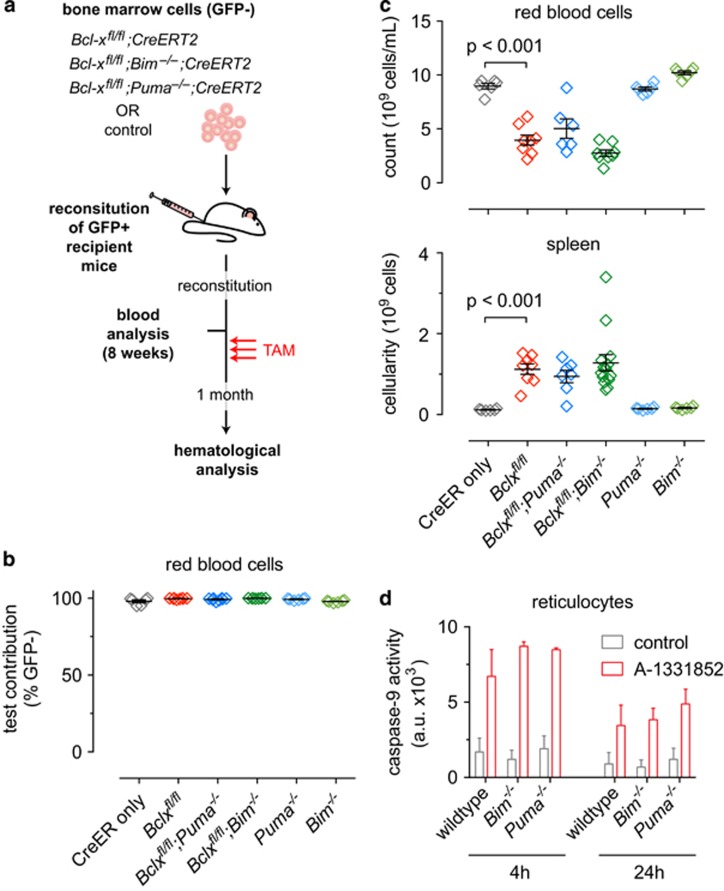
Concomitant loss of BIM or PUMA fails to rescue anaemia caused by acute deletion of *Bclx*. (**a**) Lethally irradiated GFP^+^ recipient mice were reconstituted with ‘test’ bone marrow of the indicated genotypes (GFP^−^). At 8 weeks after reconstitution a mandible bleed was taken from each mouse and analysed to confirm successful haematopoietic reconstitution and to establish baseline blood cell counts. Mice were then treated with tamoxifen (TAM, red arrows). Peripheral blood and spleens were collected for analysis 1 month after TAM administration. (**b**) Test red blood cell contribution at 8 weeks, prior to TAM treatment. *n*=6–8 (*N*=3–4 bone marrow donors); mean±S.E.M shown. (**c**) Red blood cell counts and spleen cellularity after TAM treatment. *n*=6–14 (*N*=3–7 bone marrow donors); mean±S.E.M. (**d**) *In vitro* Caspase-9 activity in FACS-sorted reticulocytes, control *versus* A-1331852-treated (1 *μ*M). a.u. denotes arbitrary units. Significant differences as shown, determined by unpaired Student’s *t*-test

We next investigated which pro-apoptotic BH3-only protein might be critical for reticulocyte apoptosis following loss of BCL-XL. BIM and PUMA are prime candidates because they bind to all pro-survival BCL-2 proteins and are critical for apoptosis induction in many haematopoietic cell types, particularly lymphocytes,^20, 42, 43, 44^ after exposure to diverse cytotoxic insults.^22, 45, 46^ To investigate the roles of BIM and PUMA, we reconstituted lethally irradiated mice with bone marrow from *Bclx*^*fl/fl*^*;RosaCreERT2*^*Ki/+*^*;Bim*^*−/−*^, *Bclx*^*fl/fl*^*;RosaCreERT2*^*Ki/+*^*;Puma*^*−/−*^ or relevant control mice (Figure 4a). Mice expressing GFP in all cells, importantly including erythroid cells,^47^ were used as recipients to distinguish donor-derived haematopoietic cells (GFP^−^) from residual recipient-derived cells (GFP^+^) (Supplementary Figure 5).

Efficient haematopoietic reconstitution was confirmed 8 weeks post-transplantation with donor-derived (GFP^−^) red blood cells present at >95% (Figure 4b). *Bclx* gene deletion was induced with tamoxifen and after 1 month bone marrow, spleen and peripheral blood cells were analysed. As expected,^26^ tamoxifen-treated *Bclx*^*fl/fl*^*;RosaCreERT2*^*Ki/+*^ reconstituted mice developed severe anaemia evident by low red blood cell numbers and splenomegaly, whereas *RosaCreERT2*^*Ki/+*^ reconstituted mice presented with normal blood counts and spleen cellularity (Figure 4c). Surprisingly, all tamoxifen-treated *Bclx*^*fl/fl*^*;RosaCreERT2*^*Ki/+*^*;Bim*^*−/−*^reconstituted mice presented with splenomegaly and anaemia (Figure 4c). Hence, in contrast to its importance in embryonic erythropoiesis, loss of BIM failed to mitigate the erythropoietic insufficiency caused by BCL-XL deletion in adult mice. Loss of PUMA also provided no protection. Accordingly, no differences in caspase-9 activity were observed between reticulocytes from wild-type, *Bim*^*−/−*^ or *Puma*^*−/−*^ mice following treatment with the BCL-XL-selective inhibitor A-1331852 (Figure 4d).

These findings show that on their own BIM and PUMA are not essential for the loss of reticulocytes and anaemia caused by BCL-XL deletion.

### PUMA has a minor role in anaemia induced in adult mice by acute BCL-XL loss

**Figure 5:**
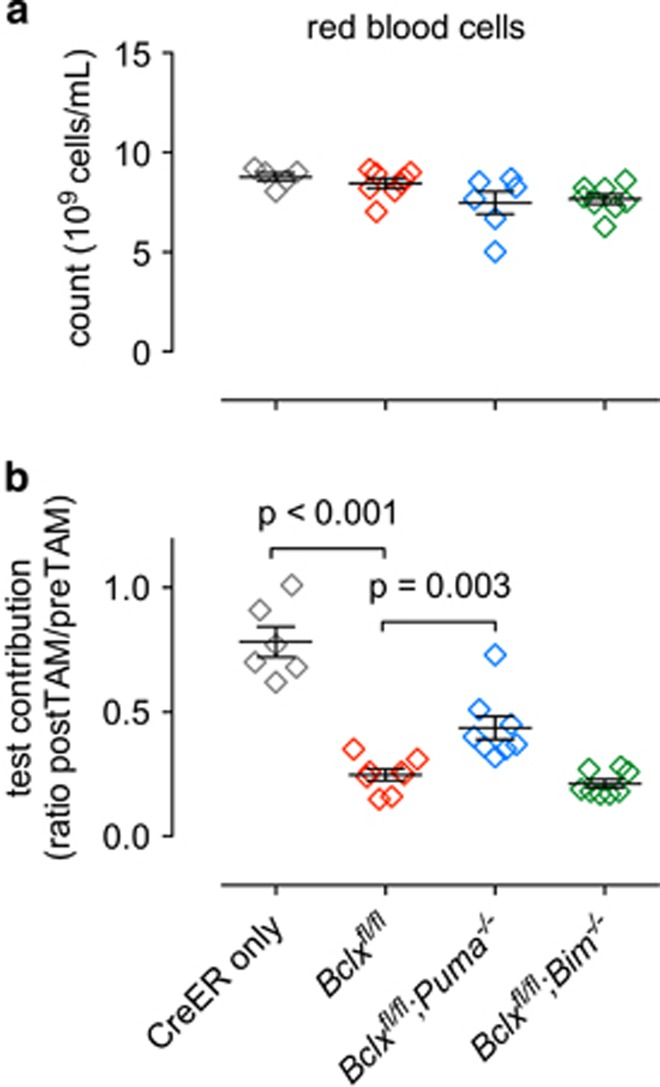
Loss of PUMA partially alleviates impaired erythropoiesis caused by acute deletion of *Bclx*. Lethally irradiated GFP^+^ mice (2 × 5.5 Gy) were reconstituted with a 1-to-1 mixture of bone marrow cells from wild-type mice (‘competitor’ GFP^+^) and mice of the indicated genotypes (‘test’ GFP^−^). Eight weeks after reconstitution blood was taken from reconstituted mice and analysed for test *versus* competitor contribution. *Bclx* deletion was then induced by administering tamoxifen. Mice were killed 1 month after tamoxifen administration and peripheral blood was collected for analysis. (**a**) Overall total red blood cell counts including both GFP^+^ and GFP^−^ populations. *n*=5–8 (*N*=3–4 bone marrow donors); mean±S.E.M. (**b**) Ratio of test (GFP^−^) contribution to red blood cell population pre- and post-tamoxifen treatment. *n*=5–8 (*N*=3–4 bone marrow donors); mean±S.E.M. Significant differences as shown, determined by unpaired Student’s *t*-test

To further investigate whether BIM or PUMA may have a minor role in the apoptosis of erythroid cells caused by BCL-XL loss, we adopted a mixed bone marrow reconstitution approach (Figure 4a). GFP^+^ mice were reconstituted with a 1:1 mix of bone marrow cells comprising wild-type (GFP^+^) ‘competitor’ cells and ‘test’ cells of the following genotypes: *RosaCreERT2*^*Ki/+*^, *Bclx*^*fl/fl*^*;RosaCreERT2*^*Ki/+*^, *Bclx*^*fl/fl*^*;RosaCreERT2*^*Ki/+*^*;Bim*^*−/−*^ and *Bclx*^*fl/fl*^*;RosaCreERT2*^*Ki/+*^*;Puma*^*−/−*^ (all GFP^−^). As expected, the presence of the wild-type cells prevented the anaemia caused by BCL-XL deletion (Figure 5a). When the ratio of test to competitor derived cells was determined within the red blood cell compartment, relative to the *RosaCreERT2*^*Ki/+*^ control cells, the *Bclx*^*fl/fl*^*;RosaCreERT2*^*Ki/+*^ cells were profoundly depleted (Figure 5b). This deficit was mildly alleviated by concomitant loss of PUMA, whereas loss of BIM had no impact (Figure 5b). These results indicate that PUMA has a minor role in the loss of red blood cells resulting from acute BCL-XL loss.

## Discussion

Throughout life large numbers of red blood cells must be continuously produced in the bone marrow to offset their ongoing loss in the periphery. The high rate of proliferation of progenitors and the marked changes in cellular architecture during differentiation must impose stresses that activate apoptosis signalling. Thus, inhibitors of apoptosis are expected to be critical to sustain survival of differentiating erythroid cells and previous studies identified BCL-XL as the critical factor. Germline deficiency for BCL-XL causes embryonic lethality around E13.5 owing to aberrant death of erythroid and neuronal cells.^24^ Loss of BIM was shown to reduce the excess apoptosis of haematopoietic fetal liver cells in *Bclx*^*−/−*^ mice (although this did not prevent embryonic lethality).^25^ Studies *in vitro* concluded that BCL-XL is needed for the survival of late-stage erythroid progenitors.^30, 48^ However, the precise stage during erythropoiesis at which BCL-XL is critical for survival was not resolved.

Cell type-specific conditional *Bclx* gene deletion in adult mice caused severe anaemia, accompanied by splenomegaly and elevated numbers of erythroblasts. This led to the conclusion that BCL-XL is only essential during terminal erythroid maturation. Because of the elevated reticulocyte numbers, the authors of that study attributed the anaemia to excessive haemolysis.^26^ Conversely, a subsequent study using the same model, but a later time point for analysis, reported reduced reticulocyte numbers. The authors of the second study concluded that an erythrocyte production defect was responsible for the anaemia caused by loss of BCL-XL and proposed that mature erythroblasts rather than reticulocytes depend on BCL-XL for survival.^31^

Using genetic and pharmacological approaches coupled with FACS analysis to delineate discrete stages of erythroid differentiation,^36^ we found that BCL-XL is essential for reticulocyte survival but largely dispensable for the survival of orthochromatic erythroblasts and earlier progenitors. This is consistent with reports that the highest levels of BCL-XL are observed during terminal erythrocyte differentiation when haemoglobin production is maximal.^8^ Apoptosis of reticulocytes following acute *Bclx* gene deletion – documented by appearance of apoptosis-specific markers – results in a compensatory increase in erythroblasts in the bone marrow and the spleen. Our findings clarify previously reported observations. Our use of the *RosaCreERT2* allele enabled efficient inducible *Bclx* gene deletion resulting in anaemia within 1 month, compared with the 3–4 months in the previously described models ^26^. This allowed primary effects of BCL-XL loss to be studied in the absence of potentially confounding secondary effects or compensatory processes. Our acute *Bclx* deletion model closely mimics the scenario of therapeutic BCL-XL inhibition by BH3-mimetic drugs and therefore provides insight into their likely impact on patients.

Pro-apoptotic PUMA and BIM were investigated as potential initiators of reticulocyte death caused by loss of BCL-XL. In contrast to the published role for BIM as a critical initiator of erythrocyte precursor death during embryogenesis in *Bclx* deficient mice,^25^ we found that concomitant loss of BIM did not alleviate the anaemia caused by acute BCL-XL loss in the adult. Loss of PUMA also provided no rescue. However, experiments using mixed bone marrow reconstituted mice provided evidence that PUMA plays a minor role in the reticulocyte death. Hence, we conclude that other BH3-only proteins may be critical (either by themselves or together with PUMA and BIM) for the induction of apoptosis following BCL-XL loss in reticulocytes.

Our findings demonstrate that reticulocytes are the key erythroid precursors that require BCL-XL for survival and that acute loss of BCL-XL results in anaemia owing to increased apoptosis of reticulocytes and thus diminished production of mature red blood cells. In mice, mature red blood cells spontaneously lyse after 8–20 days, unless taken up by macrophages for retrieval of iron. Hence, anaemia will ensue upon loss of BCL-XL-dependent reticulocytes, although in mice this can be delayed by compensatory erythropoiesis in the spleen. This accounts for the relatively slow development of anaemia, the low numbers of reticulocytes as well as the elevated numbers of the more primitive erythroid precursors.

In humans, red blood cells survive in the circulation for up to 120 days, significantly longer than mice. The extended survival of red blood cells in humans may provide a therapeutic window for intervention with BCL-XL inhibitors, unless erythropoiesis is already compromised prior to treatment.

## Materials and Methods

### Reagents

The BCL-2 inhibitor ABT-199 (ref. 38) was provided by AbbVie and QVD-OPH was purchased from MP Biomedical (#03OPH10903, Santa Ana, CA, USA).^49^ The BCL-XL inhibitor A-1331852 was prepared according to procedures described in the literature (patent: Wang *et al.*, WO 2013055897).^38^ Bafilomycin was purchased from Sigma Aldrich (#B1793, St. Louis, MO, USA). Tamoxifen was purchased from Sigma Aldrich.

### Mice

All experiments with mice were conducted according to the guidelines of The Walter and Eliza Hall Institute of Medical Research Animal Ethics Committee. Mouse strains utilised in this study have been previously published (*Bclx*^*fl*^,^26^ *Bim*^*−/−*^,^20^ *Puma*^*−/−*^,^42^ UBC-GFP Tg^47^ and *RosaCreERT2*^37^). All mice were either generated on a C57BL/6 background or crossed onto this background for at least 20 generations before commencement of our studies. Tamoxifen administration was performed by oral gavage, 3.6 *μ*g/day on 3 consecutive days. Blood transfusion was performed with blood isolated from UBC-GFP Tg mice (GFP^+^), 250 *μ*l blood/recipient.

Genotyping was performed as previously reported.^22^ Oligonucleotide sequences for genotyping of the mutant alleles will be provided on request.

### Generation of bone marrow chimaeric mice

Haematopoietic reconstitutions were performed as previously described.^22^

### Histology and blood film analysis

Sternum and spleen specimens were fixed in 10% formalin. Paraffin tissue sections were prepared at 7.5 *μ*m and stained with haematoxylin and eosin. Images (sternum × 20; spleen × 40) were taken with an Olympus BX43 (Olympus, Shinyuku, Japan) using the acquisition program CellSens Standard (Olympus). Slides for analysis of blood cell morphology were prepared by spreading freshly venesected blood by hand onto slides, which were allowed to air-dry. Blood films were fixed in methanol and stained using May-Grunwald and Giemsa stains. Blood film images were acquired using a Nikon Eclipse 90i microscope. Images presented were taken using a × 100 oil objective and inset views for reticulocyte features utilised digital zoom.

### Serum analysis

Blood from mice was collected 1 month after tamoxifen treatment via cardiac puncture. Bleeds were left to coagulate at room temperature for 5 min and centrifuged for 1 min at 13 000 rpm to collect serum. Sera were stored at −20 °C and analysed using Architect c1600 (Abbott Diagnostics, Santa Clara, CA, USA).

### Flow cytometric analysis

Spleen and bone marrow cells were harvested and single-cell suspensions prepared. Cells were counted using the CasyCell Counter (Schaefe System GmbH, Neunkirchen, Germany). Retro-orbital bleeds were collected into EDTA for differential cell counts using an ADVIA 2120 analyser (Siemens Healthcare PTY, Ltd, Bayswater, VIC, Australia). The analysis of red blood cells and leukocytes from either wild-type or test bone marrow in the haematopoietic reconstituted mice was determined by flow cytometric analysis of GFP and staining for haematopoietic subset-specific surface markers (CD44 [IM781], TER119 [TER119], B220 [RA3-6B2] or CD19 [ID3], GR-1 [RB6-8C5], MAC-1 [MI/70], CD45.2 [5.450.15.2], CD3 [KT3-1.1]). Antibodies were conjugated to FITC, R-PE or APC. For erythroid precursor analysis, mature cells were gated out prior to CD44 *versus* FSC analysis using a ‘DUMP’ channel with the following surface markers: B220 or CD19, GR-1, MAC-1, CD45.2, CD3. GFP^+^ (wild-type bone marrow derived) and GFP^−^ (test bone marrow derived) cells were used to determine relative contributions in the haematopoietic reconstituted mice. Samples were analysed in a LSR-II flow cytometer (BD Biosciences, San Jose, CA, USA). Dead cells were excluded using forward and side light scatter plus propidium iodide exclusion (Sigma Aldrich).

For stem and progenitor cell analysis, cells were stained with Alexa Fluor 700 conjugated antibodies against the following surface markers: CD45R (B220), CD19, GR-1, Ly6G, F4/80, TER119, NK1.1, CD2, CD4, and CD8 and CD117 PerCPCy5.5 (c-KIT, clone 2B8 BD Biosciences), SCA-1 Alexa Fluor 594, CD135 PE (FLT3/FLK2 clone A2F10 Biolegend, San Diego, CA, USA), CD34 Alexa Fluor 647 (clone RAM34 BD Biosciences) CD16/32 biotin (Fc*γ* receptor II/III clone 24G2 BD Biosciences) and Streptavidin BV650 (BD Biosciences) and analysed in a Fortessa I flow cytometer (BD Bioscience). Dead cells were excluded using forward and side light scatter and Fluoro-Gold (Fluorochrome LLC, Denver, CO, USA) exclusion. Mature cells were gated out using the Alexa Fluor 700 (Lineage) ‘DUMP’ channel.

### Mitochondrial content analysis

Cells were incubated with 500 nM MitoTracker Deep Red for 20 min at 37 °C (Thermo Fisher, #M22426). Cells were then stained for the appropriate lineage markers, TER119 and CD44 to delineate specific erythroid cell populations by flow cytometry.

### Caspase-9 activity assay

5 × 10^4^ bone marrow cells were cultured for 4 or 24 h with or without the BCL-XL inhibitor A-1331852 in a 96-well plate in 100 *μ*l of medium. Plates were allowed to equilibrate at room temperature prior to adding 100 *μ*l Caspase-9 Glo reagent (Promega, Madison, WI, USA). Assays were analysed after 30 min incubation according to the manufacturer’s instructions.

### Clonogenic assays

CFU-e were enumerated by culturing single-cell suspensions of bone marrow (25 000) or spleen (50 000) in 1 ml of MethoCult 3234 medium (Stem Cell Technologies, Vancouver, BC, Canada) supplemented with 2 U/ml erythropoietin (EPO, Janssen-Cilag Ltd, Buckinghamshire, UK) with incubation in 5% CO_2_ in air for 2–3 days. The numbers of myeloid colony-forming cells in single-cell suspensions of bone marrow (25 000) or spleen (50 000) were assessed in 1 ml cultures of 0.3% agar in Dulbecco/s modified Eagles medium containing 20% newborn calf serum, stem cell factor (SCF, 100 ng/ml, WEHI), EPO (2 U/ml, Janssen-Cilag Ltd) and interleukin-3 (IL-3, 10 ng/ml). Cultures were incubated at 37 °C for 7 days in 10% CO_2_ in air. Cultures were fixed, dried onto glass slides and stained for acetylcholinesterase followed by Luxol fast blue and haematoxylin to determine the numbers and type of colonies.

### Cell survival assays *in vitro*

Bone marrow cells were collected and stained with antibodies against TER119 (TER119) and CD44 (IM781) and a lineage marker cocktail (CD3 [KT3], MAC-1 [MI/70], GR-1 [RB6-8C5], B220 [RA3-6B2], CD45.2 [5.450.15.2]). Lineage^−^TER119^+^ cells were sorted using the Aria cell sorter (BD) and further subdivided based on CD44 staining and forward light scatter. Reticulocytes and orthochromatic reticulocytes were resuspended in Dulbecco’s medium (Invitrogen, Life Technologies, Carlsbad, CA, USA) supplemented with 10% FCS (Bovogen, East Keilor, VIC, Australia), 100 mM asparagine (Sigma Aldrich) and 50 mM 2-mercapoethanol (Sigma Aldrich) without addition of cytokines. Cells were treated either with DMSO (control; 1 × 10^−4^% Sigma Aldrich), 1 *μ*M of A-1331852 (provided by AbbVie, North Chicago, IL, USA), 1 *μ*M A-1331852+25 nM QVD-OPH (MP Biomedicals) or 1 *μ*M ABT-199. Cells were collected after 0, 24, 48 and 72 h and viability was determined by FACS analysis using forward by side light scatter profile to gate for live cells or Annexin V staining.

### Statistical analysis

Cellularity, weight, blood parameters, flow cytometric results and *in vitro* cell survival were plotted and analysed with GraphPad Prism (GraphPad Software Inc, La Jolla, CA, USA). Statistical comparisons were conducted in a pair-wise manner using unpaired two-tailed Student’s *t*-test assuming equal variance. For Figure 2c we utilised the Sidak-Bonferroni method for multiple comparisons correction. For Figure 4 we performed two-way ANOVA analysis with Tukey’s multiple comparisons test. Stars indicate significant values. Error bars are presented as standard error of mean (±S.E.M.).

## Figures and Tables

**Table 1:** Haematopoietic potential determined by colony formation in semi-solid agar

	**Number of colonies per 25 000 cells**						
**Bone marrow**	**Total**	**Blast**	**G**	**GM**	**M**	**Eo**	**Meg**
*Bclx*^*fl/fl*^ (*n*=6)	106±29	14±5	24±9	23±4	12±4	7±2	21±6
*Bclx*^*fl/fl*^*;CreRERT2* (*n*=4)	114±21	21±4	26±7	24±8	14±4	7±4	23±5
	**Number of colonies per 50 000 cells**						
**Spleen**	**Total**	**Blast**	**G**	**GM**	**M**	**Eo**	**Meg**
*Bclx*^*fl/fl*^ (*n*=6)	9±1	1±1	1±1	1±1	1±1	0	6±1
*Bclx*^*fl/fl*^*;CreRERT2* (*n*=4)	21±12	5±3	3±3	2±2	4±2	1±1	7±3

Bone marrow cells were cultured in semi-solid agar in the presence of SCF+IL-3+EPO for 7 days. The numbers and types of colonies were scored from dried, stained cultures. Means±S.D.s are shown.
