# Supplementary figures and images for: The BH3-only proteins BIM and PUMA are not critical for the reticulocyte apoptosis caused by loss of the pro-survival protein BCL-XL
Source: Cell Death Dis. 2017 Jul 6;8(7):e2914–. doi: 10.1038/cddis.2017.304 (PMC5550852; doi:10.1038/cddis.2017.304)

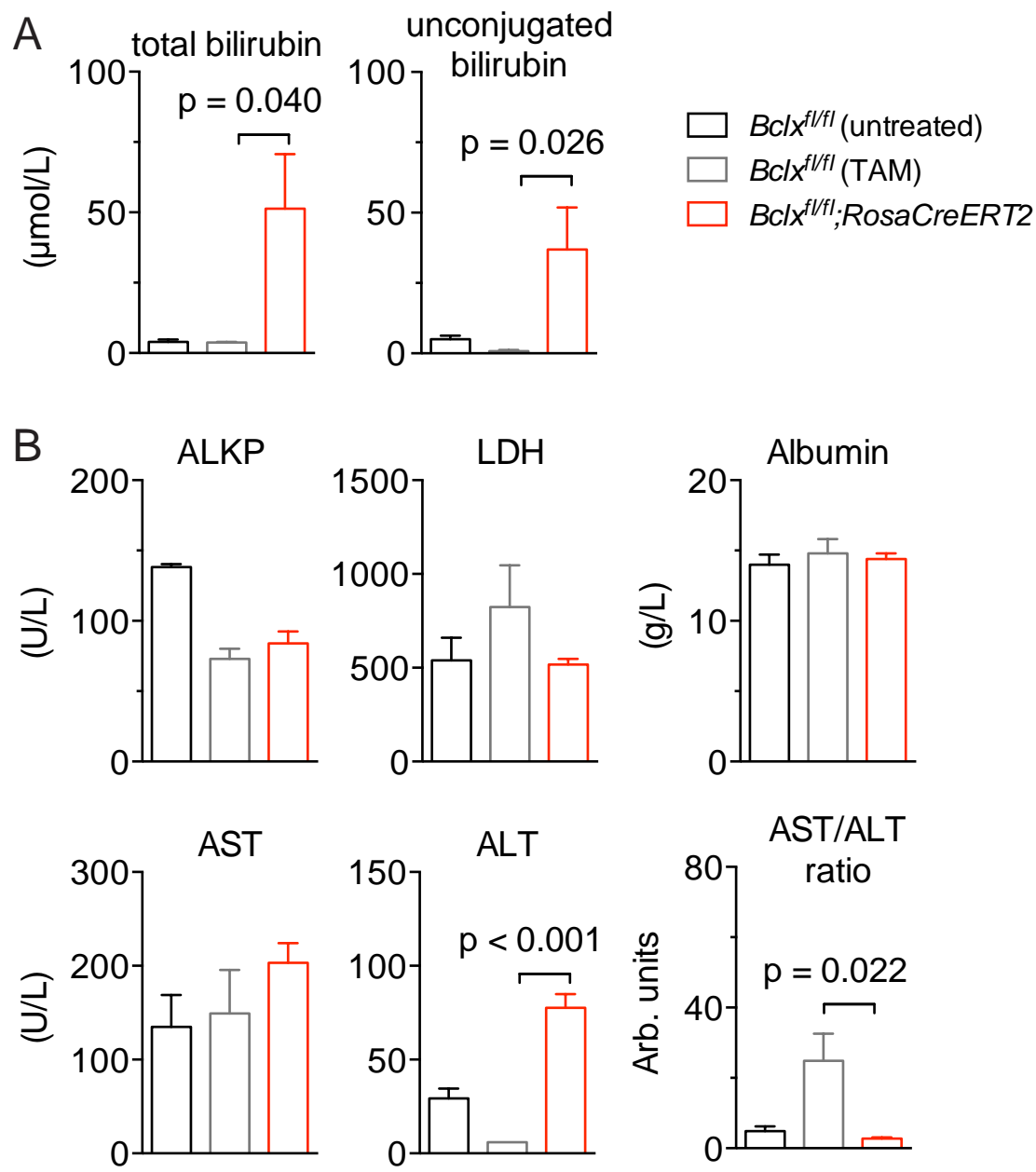

## Day 2

RosaCreERT2

*Bcl<sup>x<sup>fl/fl</sup></sup>*;RosaCreERT2

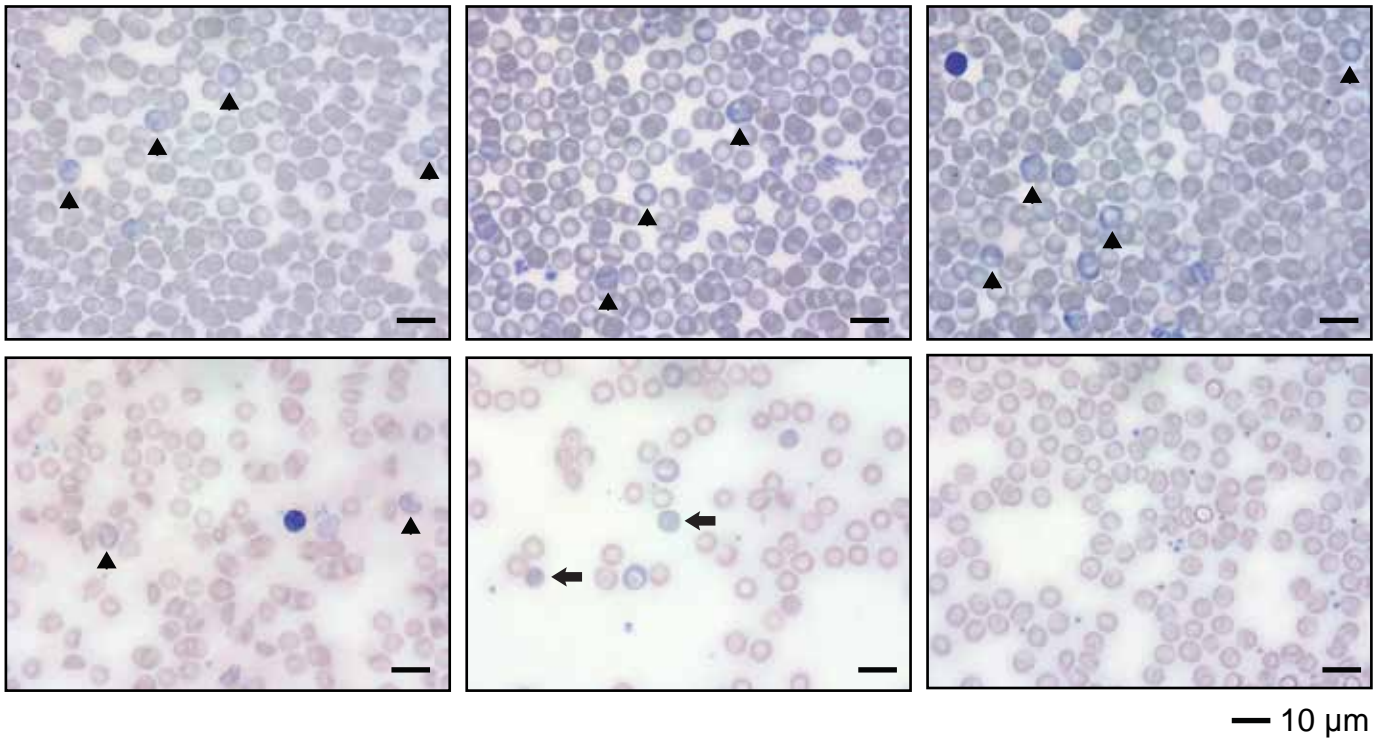

B

normal reticulocyte

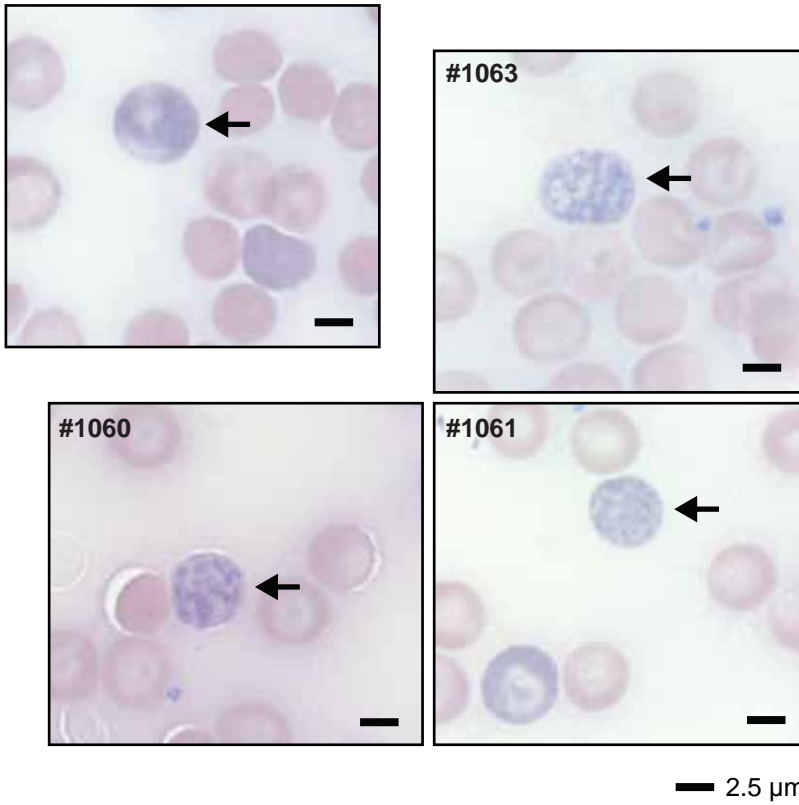

C

red blood cells

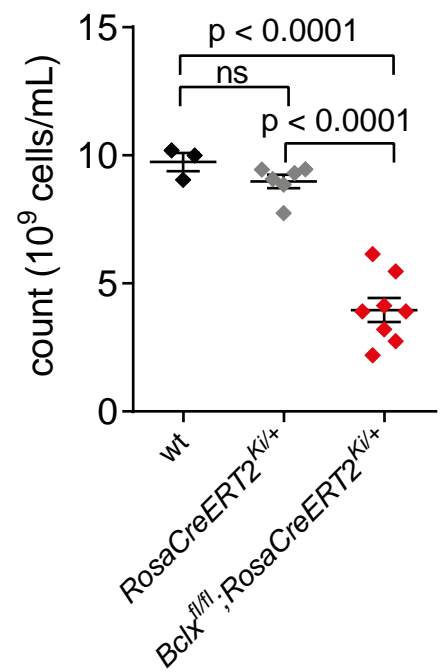

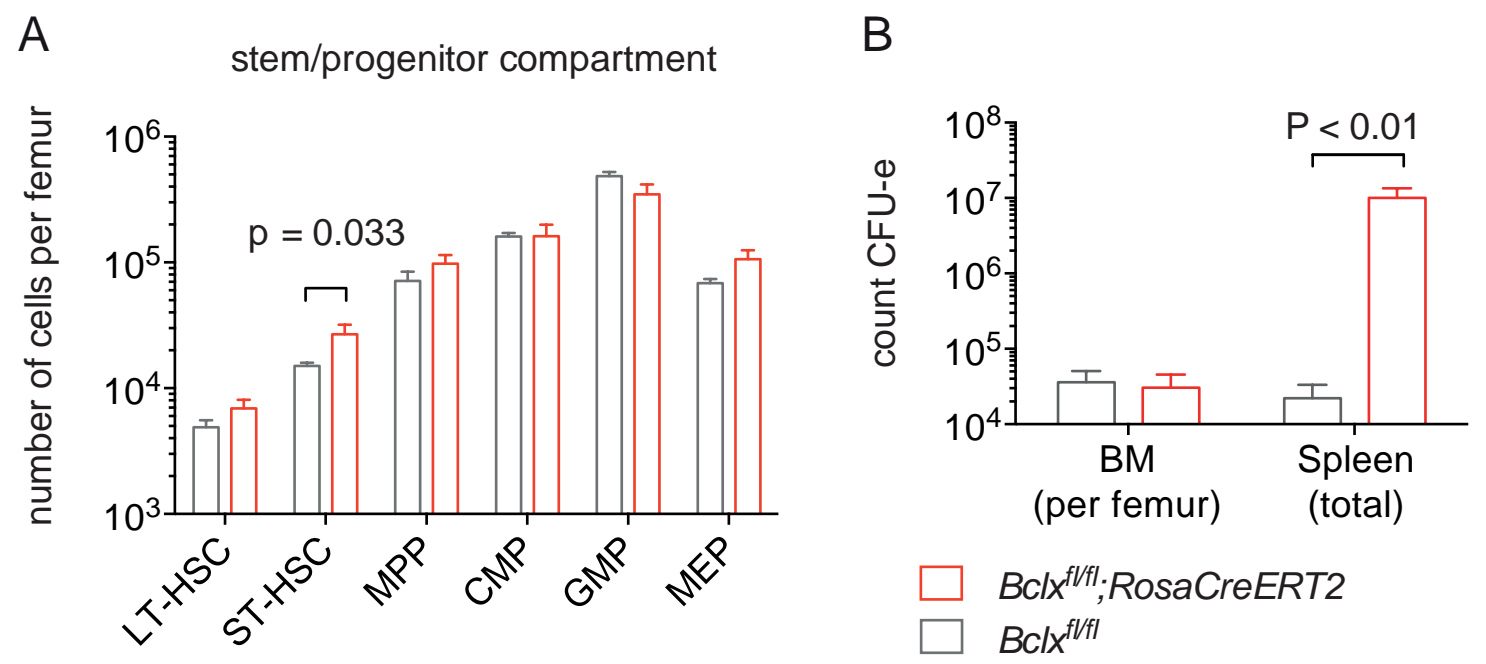

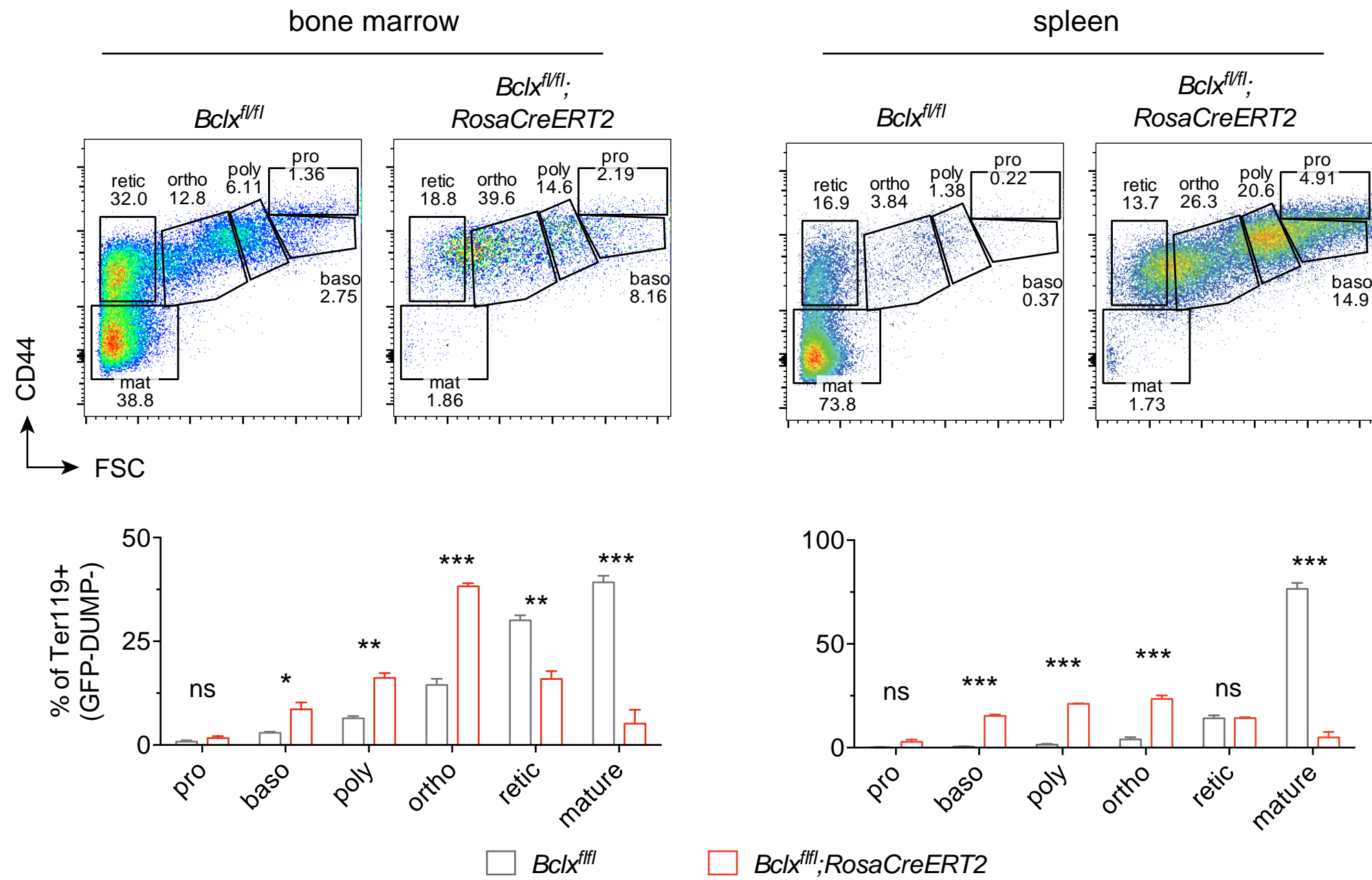

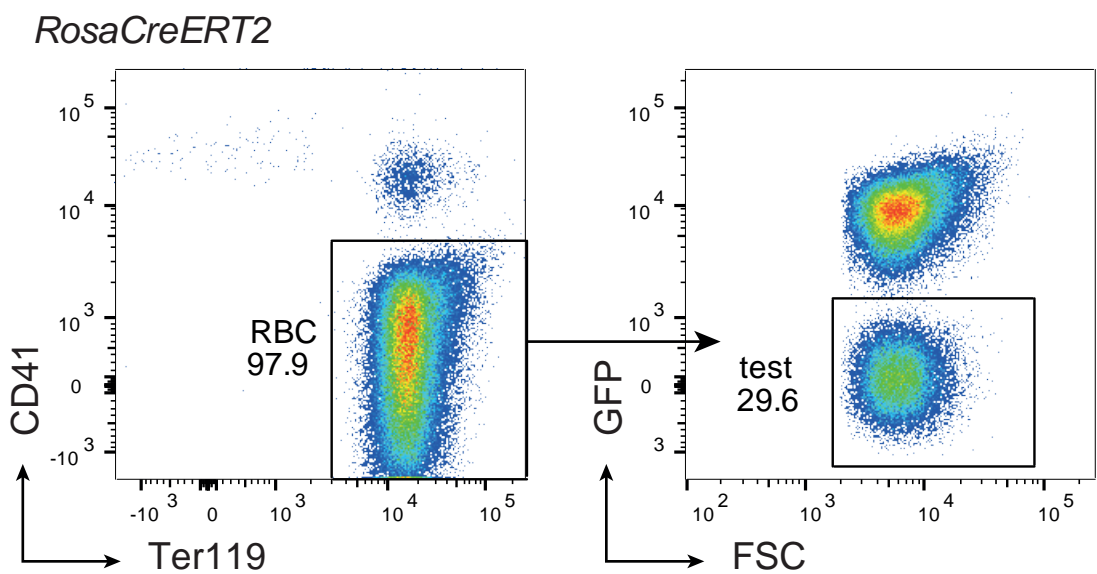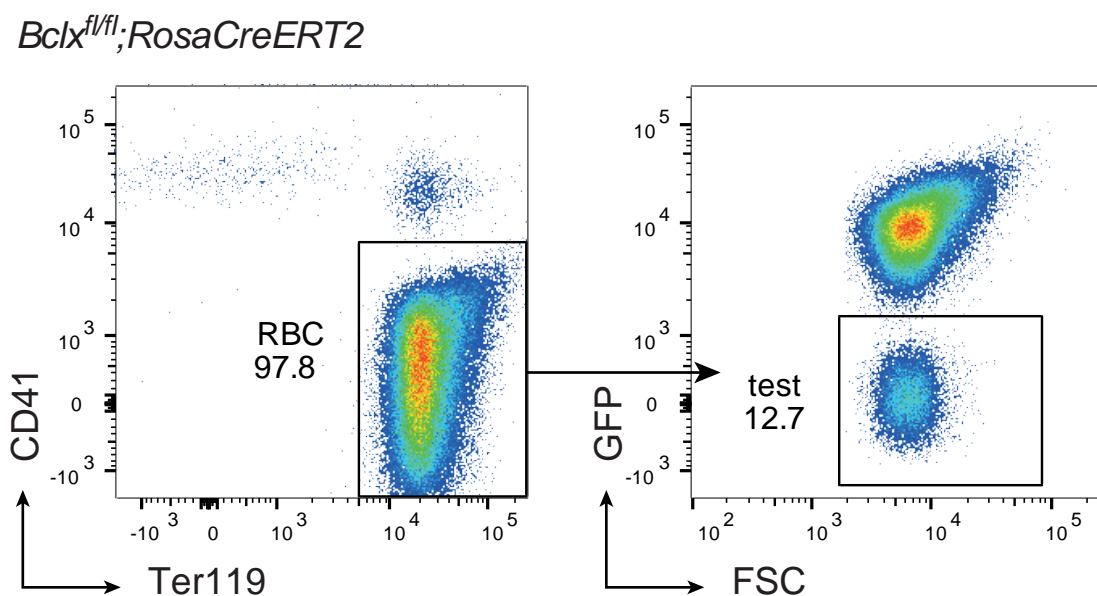

Supplement: Supplementary Figure 1 [file cddis2017304x1.pdf]
