# Supplementary material for: The BH3-only proteins BIM and PUMA are not critical for the reticulocyte apoptosis caused by loss of the pro-survival protein BCL-XL
Source: Cell Death Dis. 2017 Jul 6;8(7):e2914–. doi: 10.1038/cddis.2017.304 (PMC5550852; doi:10.1038/cddis.2017.304)
Supplement: Supplementary Figures [file cddis2017304x2.docx]

**Supplemental Figures**

**Supplemental Figure 1: Liver function is unperturbed by loss of BCL-XL.** *Bclx^fl/fl^;RosaCreERT2^Ki/+^* mice (or control *Bclx^fl/fl^* mice) were treated with tamoxifen (TAM) to induce *Bclx* gene disruption, after 1 month serum analysis of (A) total bilirubin and unconjugated bilirubin levels, as well as (B) alkaline phosphatase (ALKP), lactate dehydrogenase (LDH), albumin, aspartate aminotransferase (AST) levels, and alanine aminotransferase (ALT) levels was performed. n = 4-6. Data are presented as mean ± SEM. Significant p values are shown, unpaired Students t test.

**Supplemental Figure 2: Acute deletion of *Bclx* results in loss of circulating reticulocytes in the blood.** (A) May-Grunwald and Giemas staining of blood films from RosaCreERT2 or *Bclx^fl/fl^;RosaCreERT2^Ki/+^* mice on Day 0, 1 and 2 following tamoxifen administration. (B) Comparison of the morphology of a normal reticulocyte vs reticulocytes in which *Bclx* has been acutely deleted. Images were taken using a 100X oil objective, the bar in (A) represents 10 µm and in (B) 2.5 µm. Number corresponds to mouse nunber. (C) Lethally-irradiated GFP^+^ mice (2 x 5.5 Gy) were reconstituted with bone marrow cells harvested from wildtype, *RosaCreERT2^Ki/+^*, or *Bclx^fl/fl^;RosaCreERT2^Ki/+^* mice. Eight weeks after reconstitution Cre activation was induced with tamoxifen. Mice were sacrificed 1 month after tamoxifen administration and peripheral blood was analysed to determine red blood cell counts. n = 3-8; mean ± SEM. Significant differences with P values as shown, determined by unpaired Students t test.

**Supplemental Figure 3: Acute deletion of BCL-XL results in elevated erythroid progenitor production.** *Bclx^fl/fl^;RosaCreERT2^Ki/+^* mice (or control *Bclx^fl/fl^* mice) were treated with tamoxifen (TAM) to induce *Bclx* gene deletion, and analysis of bone marrow and spleen was performed after 1 month. (A) Identification and enumeration of stem/progenitor cells: LT-HSC (Lineage^‑^ Sca1^+^ cKit^+^ CD34^‑^ CD135 [Flt3/Flk2]^‑^), ST-HSC (Lineage^‑^ Sca1^+^ cKit^+^ CD34^+^ CD135 [Flt3/Flk2]^‑^), MPP (Lineage^‑^ Sca1^+^ cKit^+^ CD34^+^ CD135 [Flt3/Flk2]^+^), CMP (Lineage^‑^ Sca1^‑^ cKit^+^ CD34^+^ CD16/32 [FcγR II/III]^‑^), GMP (Lineage^‑^ Sca1^‑^ cKit^+^ CD34^+^ CD16/32 [FcγR II/III]^+^), MEP (Lineage^‑^ Sca1^‑^ cKit^+^ CD34^‑^ CD16/32 [FcγR II/III]^‑^). (B) CFU-e enumeration in the spleen and the bone marrow as determined by colony formation assay. Data are presented as mean ± SEM. Significant p values are shown.

**Supplemental Figure 4: BCL-XL-deficient bone marrow cells poorly reconstitute erythropoiesis following transplantation**

Bone marrow cells harvested from *Bclx^fl/fl^;RosaCreERT2^Ki/+^* mice (or control *Bclx^fl/fl^* mice) 4 weeks after tamoxifen treatment were used to reconstitute lethally-irradiated GFP^+^ recipient mice . *Bclx^fl/fl^;RosaCreERT2^Ki/+^* reconstituted mice developed anemia after 3 weeks and were transfused with 250 μL GFP^+^ blood. Flow cytometric analysis of erythroid lineage cells in the bone marrow and spleen at 4 weeks post-reconstitution (1 week post-transfusion with GFP^+^ blood cells), with quantitation. Plots gated to display DUMP^-^Ter119^+^GFP^-^ cells (i.e. transfused GFP^+^ cells excluded): pro-erythroblasts (pro), basophilic erythroblasts (baso), polychromatic erythroblasts (poly), orthochromatic erythroblasts (ortho), reticulocytes (retic), and mature red blood cells (mature). Data representative of n = 3 mice per genotype. Significant differences as indicated, unpaired Students t test, adjusted for multiple testing (Holm-Sidak); * p < 0.05, ** p < 0.01, *** p < 0.001.

**Supplemental Figure 5: UBC-GFP expression allows robust discrimination of GFP+ and GFP- red blood cells**. Lethally-irradiated GFP^+^ mice (2 x 5.5 Gy) were reconstituted with bone marrow cells harvested from UBC-GFP Tg mice (wildtype; ‘competitor’) mixed with either *RosaCreERT2^Ki/+^*, or *Bclx^fl/fl^;RosaCreERT2^Ki/+^* (‘test’) bone marrow. Eight weeks after reconstitution tamoxifen was administered to activate Cre-induced *Bclx* deletion. Mice were sacrificed 1 month after tamoxifen administration and peripheral blood was analyzed by flow cytometry to determine the relative contribution of competitor and test-derived cells to the red blood cell population. Representative FACS plots shown gated on red blood cells by FSC *vs.* SSC profile.
